# The Effect of Esmolol Versus Dexmedetomidine on Postoperative Pain Control in Endoscopic Sinus Surgery: A Randomized Trial

**DOI:** 10.5812/aapm-158065

**Published:** 2025-05-26

**Authors:** Amany Faheem Omara, Alaa Mohammed Abo hagar, Asmaa Fawzy Amer

**Affiliations:** 1 Department of Anaesthesia and Surgical Intensive Care, Faculty of Medicine, Tanta University, Tanta, Egypt; 2 Lecturer of Anesthesiology and Intensive Care, Department of Anesthesiology and Intensive Care, Faculty of Medicine, Tanta University, Al Gharbia governate, Tanta, Egypt

**Keywords:** Esmolol, Dexmedetomidine, Postoperative Pain, FESS

## Abstract

**Background:**

Functional endoscopic sinus surgery (FESS) is the cornerstone of treatment for nasal pathology.

**Objectives:**

This randomized study compares the ability of preoperative and intraoperative esmolol and dexmedetomidine to induce postoperative analgesia and sedation.

**Methods:**

Seventy ASA I and II patients, of either sex, scheduled for FESS, were divided into two groups: The esmolol group (group E) received an intravenous bolus dose of 0.5 mg/kg prior to the induction of anesthesia, followed by 0.05 mg/kg/min and stopped immediately upon extubation, while the dexmedetomidine group (group D) received 1 µg/kg of dexmedetomidine over 10 minutes, immediately before the induction of anesthesia, followed by a 0.5 µg/kg/hour infusion after induction and stopped immediately upon extubation. Mean arterial pressure and heart rate were monitored before induction, before and after intubation, and then every 5 to 30 minutes, as well as every 10 minutes until 90 minutes following the commencement of the IV medication infusion. The sedation level was assessed using the Ramsay sedation scale at 15, 30, and 60 minutes postoperatively. Pain scores were evaluated in the recovery room (on arrival and then 15 minutes, 30 minutes, and 1 hour later) and at 2 hours, 6 hours, 12 hours, and 24 hours. The length of the procedure, the degree of bleeding during the intervention, and the occurrence of any adverse effects were documented. Categorical data were summarized as counts and percentages and compared by the chi-square test. Continuous data were assessed for normality using the Shapiro-Wilk test. The Student’s *t*-test was used for quantitative variables that are normally distributed, whereas the Mann-Whitney test was used for quantitative variables that are not.

**Results:**

According to our findings, both esmolol and dexmedetomidine were safe and beneficial in reducing blood loss during FESS, promoting optimal surgical field quality, and improving surgical field visibility. Dexmedetomidine was far more effective in providing postoperative sedation, reducing the need for opioids, and delaying the initial need for postoperative analgesia.

**Conclusions:**

It was discovered that esmolol and dexmedetomidine both provided superior surgical field, less nasal hemorrhage, and more successful results. Dexmedetomidine caused effective sedation and a reduced need for analgesics.

## 1. Background

Functional endoscopic sinus surgery (FESS) is a minimally invasive procedure that restores sinus function and nasal cavity airflow to alleviate rhinosinusitis symptoms (1). Due to the small surgical field, minor bleeding may affect the visibility of the surgical field. To address this issue, hypotensive anesthesia is always recommended to facilitate surgical dissection (2). Dexmedetomidine is an α2-adrenergic receptor agonist with sedative, anti-sympathetic, anxiolytic, hypnotic, and analgesic effects (3). Dexmedetomidine is primarily used as an antihypertensive agent in FESS by activating central receptors, resulting in decreased norepinephrine release, blood pressure, and heart rate (4). Dexmedetomidine is considered a non-opioid analgesic with opioid-sparing effects (5). Esmolol is an ultrashort-acting selective β1-adrenergic receptor blocker that acts as an antihypertensive drug by constricting arterioles and precapillary sphincters, thereby reducing extravasation at the site of surgery (6). Esmolol also has analgesic properties and attenuates adrenergic responses, so it may reduce stress during intubation and extubation (7). Preoperative administration of esmolol may reduce the need for intraoperative intravenous and inhaled anesthetics and reduce the need for postoperative opioids (8). A meta-analysis showed that intraoperative administration of esmolol reduced intraoperative and postoperative opioid consumption (9). 

## 2. Objectives

Most studies have used esmolol and dexmedetomidine to achieve hypotensive anesthesia, but the aim of this study was to investigate the effects of esmolol and dexmedetomidine on opioid (morphine) consumption within 24 hours after surgery and pain control after FESS.

## 3. Methods

This study is a prospective randomized clinical trial conducted at Tanta University Hospital after approval by the hospital ethics committee (No. 36019/11/22) and registration on ClinicalTrials.gov on October 1, 2023 (ID: NCT05703048). Informed consent was obtained from the patients. This study was conducted from November 2022 to April 2023 on 70 ASA I and II male and female patients older than 18 years and scheduled to undergo FESS at the Department of Otolaryngology, Tanta University Hospital. Patients were excluded from the study if they had diabetes, coagulopathy, renal and hepatic dysfunction, cerebrovascular disease, cardiovascular problems, bronchial asthma, hypotension, bradycardia, allergy to dexmedetomidine or esmolol, pregnancy, or chronic drug abuse. During the preanesthetic clinic visit, the medical history of the patients was recorded, and physical examination and routine laboratory tests were performed. Patients were taught how to use the visual analog scale (VAS) (0 - 10 points, where 0 = no pain and 10 = worst pain imaginable). Upon arrival in the operating room, all participants underwent standard monitoring in the form of electrocardiogram (ECG), noninvasive blood pressure, and pulse oximetry (SpO2), after intravenous access was obtained. Patients were divided into two groups (35 patients each) using a computer-generated random number table in a 1:1 ratio: A dexmedetomidine group (group D) and an esmolol group (group E). A closed, opaque envelope was used to conceal the details of group allocation. The anesthesiologist prepared the medications and opened the envelopes accordingly. Both the patients and the observers were blinded to the data collected until the end of the study. All patients received the same general anesthesia: Induction with intravenous fentanyl 1 µg/kg, propofol 2 mg/kg, and rocuronium 0.6 mg/kg to facilitate endotracheal intubation. Anesthesia was maintained with 2% sevoflurane and 0.1 mg/kg rocuronium in an oxygen-air mixture as needed. In addition, end-tidal carbon dioxide levels were maintained between 32 and 35 mm Hg by mechanical ventilation. In group D, a dexmedetomidine (Percedex, Pfizer Co) dose of 1 µg/kg was prepared by dilution in 10 mL of 0.9% normal saline and infused over 10 minutes and one hour right before the induction of anesthesia. Following this, a continuous infusion of dexmedetomidine at a rate of 0.5 µg/kg/hour was initiated with a syringe pump after induction and stopped immediately upon extubation. For patients assigned to group E, esmolol (esmolol hydrochloride, Baxter Co) was administered as an intravenous bolus of 0.5 mg/kg, added to 10 mL of 0.9% saline before induction of anesthesia. Subsequently, an esmolol infusion was started at a rate of 0.05 mg/kg/min, delivered via a syringe pump, and ceased immediately upon extubation. Throughout the procedure, the mean arterial pressure (MAP) and heart rate (HR) were monitored at various intervals: Before induction (baseline), prior to intubation, immediately after intubation, every 5 minutes for the first 30 minutes, and then every 10 minutes up to 90 minutes after beginning the intravenous drug infusion. The goal was to maintain MAP values above 55-60 mm Hg and HR between 45 and 50 beats/min. Whenever the MAP dropped, 5 mg of intravenous ephedrine was administered; if additional doses were required, the patient was excluded from the study. Bradycardia was addressed with 0.5 mg of atropine. Once the surgical procedure was completed, the residual neuromuscular block was reversed using neostigmine (0.05 mg/kg IV) combined with atropine (0.02 mg/kg IV).

Extubation occurred once the criteria were met, at which point the emergence time was documented. Emergence time is defined as the interval from the discontinuation of anesthetics to the first eye opening in response to a verbal command. Patients were shifted to the post-anesthesia care unit (PACU). After extubation, the sedation level was assessed using the Ramsay sedation scale at 15, 30, and 60 minutes. The scale ranges from 1 (anxious, agitated, or restless) to 6 (no response). All patients were monitored in the PACU until they achieved an Aldrete score of 9 or higher (maximum stay in recovery 2 hours). Regular analgesia was provided with intravenous acetaminophen (1 g every 6 hours). Pain scores were recorded upon arrival at the PACU and at 15 minutes and 30 minutes, as well as after 2, 6, 12, and 24 hours after surgery. If the score on the VAS for pain exceeded 4, a dose of 0.1 mg/kg morphine IV was administered, with the total morphine dose calculated in the first 24 hours post-surgery serving as the primary outcome. Secondary outcomes included the incidence of postoperative nausea and vomiting (PONV) as well as the duration of surgery and the extent of bleeding evaluated by the surgical team using Fromme's 5-point scale (10). The sample size and power analysis were conducted using the Epi-Info statistical software to ensure a 95% confidence limit and 80% study power, focusing on effective pain management. We anticipated that 10% of the best treatment group would require opioids, compared to 40% in the least favorable treatment group (11). Consequently, the calculated sample size was N = 33 for each group, and we enrolled 35 cases per group to account for potential dropouts. For data analysis, we used IBM SPSS software version 20.0 (IBM Corp, Armonk, NY). Categorical data were summarized as counts and percentages, and we employed the chi-square test to compare the two groups. Continuous data were first assessed for normality using the Shapiro-Wilk test. Quantitative data were reported as ranges (minimum and maximum), means, standard deviations, and medians. The Student’s *t*-test was used for quantitative variables that are normally distributed, whereas the Mann-Whitney test was used for quantitative variables that are not. We determined the significance of the results at the 5% level.

## 4. Results

**Figure 1.**
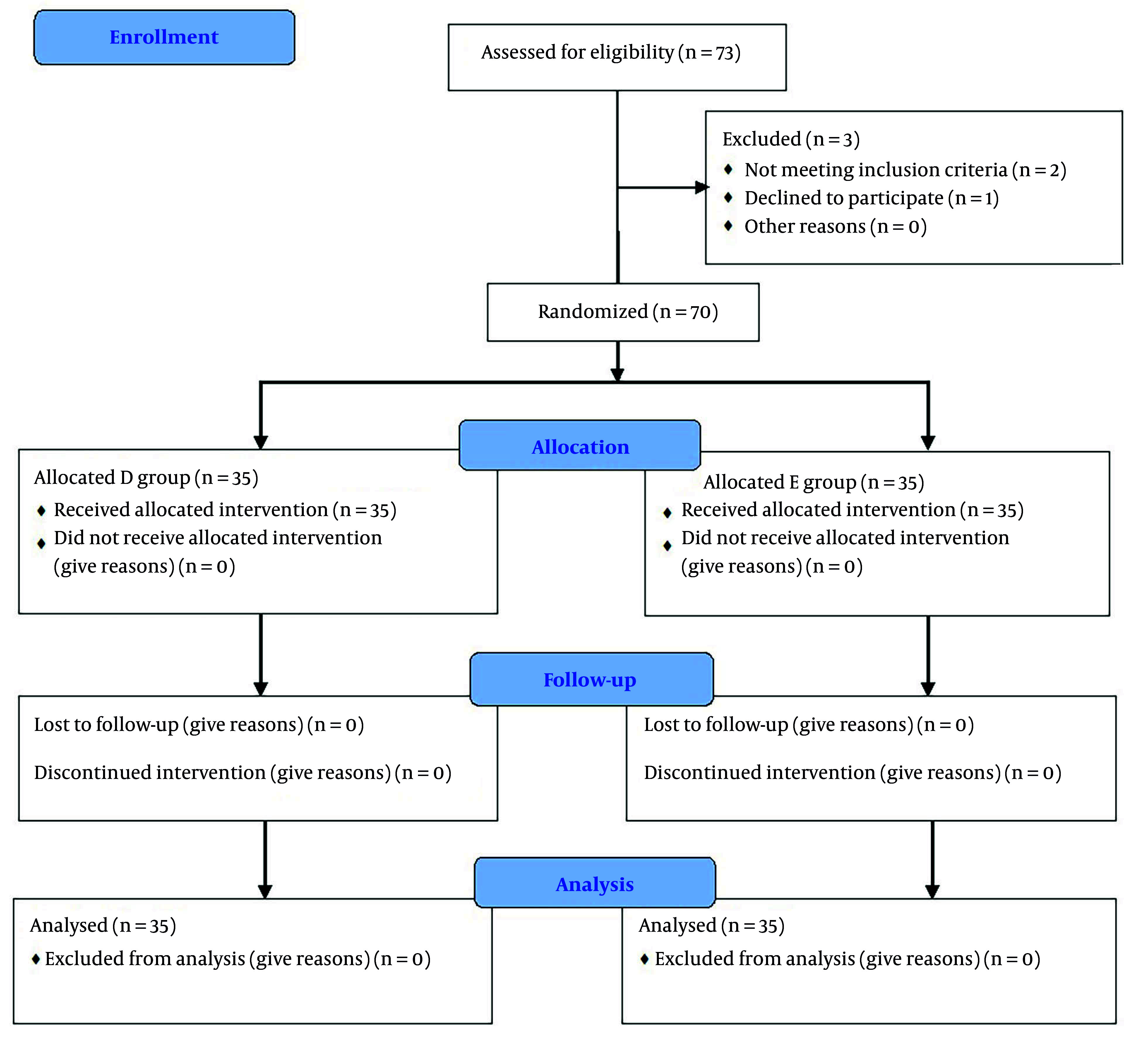
Consort flow chart

Seventy-three patients were initially deemed eligible for the study. However, one patient declined to participate, while two others were excluded—one due to sinus bradycardia and the other due to borderline blood pressure at the outset. Ultimately, seventy patients were enrolled and randomly assigned to one of two groups, with 35 patients in each (refer to Figure 1). The two groups were similar in terms of age, sex, BMI, and secondary outcomes (duration of surgery, quality of surgical field, and amount of blood loss) (Table 1). Additionally, no patient exceeded a 2-hour stay in the PACU.

**Table 1.** Comparison Between the Three Groups Studied According to Different Parameters ^a^

Variables	Dexmedetomidine Group (n = 35)	Esmolol Group (n = 35)	P-Value
**Age (y)**	31.1 ± 2.17	32 ± 2.04	0.073
**Gender**			0.803
Male	23 (65.7)	22 (62.9)	
Female	12 (34.3)	13 (37.1)	
**BMI (kg/m** ^**2**^ **)**	31.2 ± 1.98	30.7 ± 3.28	0.482
**Duration (min)**	128.9 ± 19.5	127 ± 7.68	0.592
**Ramsy**			< 0.001 ^b^
Mean ± SD	3.23 ± 0.43	1.97 ± 0.38	
Median (min - max)	3 (3 - 4)	2 (1 - 3)	
**Total morphine**	18.7 ± 3.32	20.1 ± 1.88	0.028 ^b^
**Number required rescue analgesia**	35 (100)	35 (100)	–
**Surgical field**			0.794
Good	24 (68.6)	25 (71.4)	
Very good	11 (31.4)	10 (28.6)	
**Blood loss; median (min - max)**	2 (1 - 2)	2 (1 - 2)	-
**Time of analgesia; median (min - max)**	120 (60 - 120)	30 (15 - 60)	-
**Time to extubation**			< 0.001 ^b^
Mean ± SD	8.26 ± 0.98	3.83 ± 1.01	
Median (min - max)	8 (7 - 10)	4 (2 - 5)	
**Bradycardia**	0 (0)	0 (0)	–
**PONV**	0 (0)	0 (0)	–
**Intubation response**	0 (0)	0 (0)	–
**Extubation response**	0 (0)	0 (0)	–

Abbreviations: SD, standard deviation; Min, minimum; Max, maximum; PONV; postoperative nausea and vomiting; BMI; body mass index.

^a^ Values are presented as mean ± SD or No. (%) unless otherwise indicated.

^b^ Statistically significant at P ≤ 0.05.

Each patient in the study required postoperative rescue analgesia. Notably, the total consumption of postoperative rescue morphine was significantly lower in the dexmedetomidine group, which also experienced a longer duration of analgesia compared to the esmolol group (P = 0.028 and P = 0.001, respectively). Additionally, patients in the dexmedetomidine group experienced delayed extubation and exhibited higher Ramsay sedation scores compared to those in the esmolol group (P = 0.001 for both comparisons). Importantly, no adverse events, such as significant bradycardia (less than 45 beats/min), hypotension (mean blood pressure < 50 mm Hg), and PONV, were recorded in either group (Table 1). 

**Figure 2.**
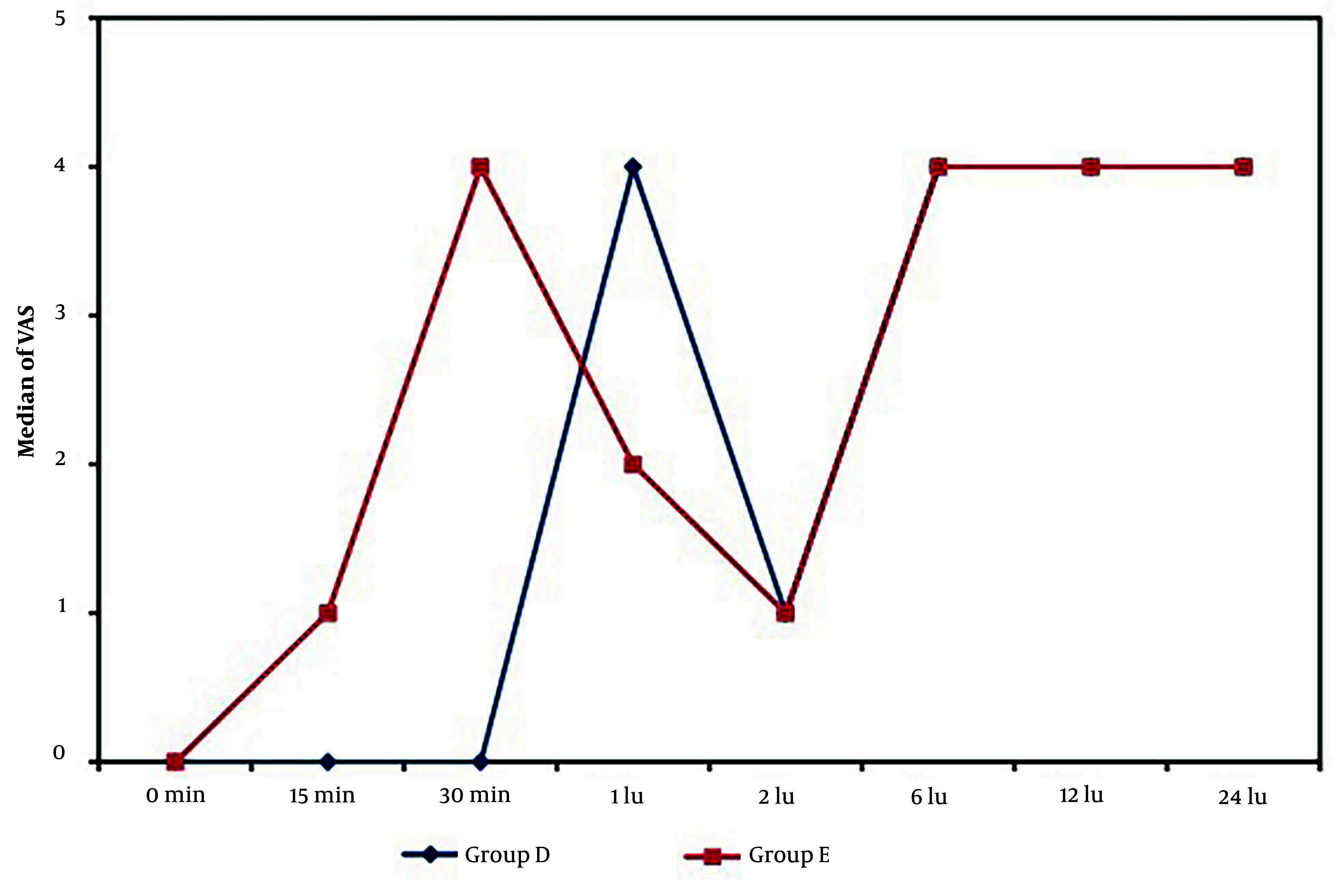
Comparison between the two groups studied according to VAS

Both groups recorded comparable VAS scores (primary outcome) at 2, 6, 12, and 24 hours postoperatively (P = 0.488, 0.529, 0.055, and 0.390, respectively). However, the VAS scores were significantly lower in the dexmedetomidine group compared to the esmolol group at 15 and 30 minutes after surgery (P = 0.001 for both). Conversely, the esmolol group demonstrated significantly lower VAS scores than the dexmedetomidine group at the 1-hour mark postoperatively (P = 0.001), as illustrated in Figure 2. 

## 5. Discussion

Our study indicated that both dexmedetomidine and esmolol proved to be effective and safe in ensuring optimal surgical field quality, enhancing visualization during FESS, and reducing blood loss. This efficacy can be attributed to the hypotensive effect of the beta blocker and dexmedetomidine, which promotes the release of norepinephrine. This raises sympathetic tone, causing arteriole and precapillary sphincter vasoconstriction, as α-adrenergic activity is unhindered. In addition, esmolol helps reduce cardiac output, thereby reducing tissue blood flow and minimizing bleeding associated with capillary damage (12). Guven et al. (13) found similar results in their study on the effects of dexmedetomidine during FESS, noting its effectiveness in ensuring a dry surgical field. Furthermore, Erbesler et al. (14) found no discernible differences between esmolol and dexmedetomidine in their ability to offer good visibility of the surgical site. In a similar study, Goksu et al. (15) examined the hemodynamic effects of dexmedetomidine administered perioperatively and found it to be effective in creating a comfortable surgical field for patients undergoing FESS. Additionally, Sabry and Elmawy (16) discovered that both dexmedetomidine and esmolol effectively optimized surgical conditions by inducing a dry surgical field that enhanced visibility and reduced operative time in pediatric patients undergoing nasal procedures.

Regarding postoperative analgesic requirements and sedation, our findings indicate that dexmedetomidine significantly improved postoperative sedation, decreased opioid consumption, and extended the time to the first request for analgesia. As a highly selective, specific, and potent α2-adrenergic agonist, dexmedetomidine provides analgesia, sedation, hypotension, and anesthetic-sparing effects when administered systemically (17). Dexmedetomidine's central action on the spinal cord's locus coeruleus and dorsal horn is primarily responsible for the sedative and postoperative analgesic effects (18). By intensifying the effects of opioids, α2 receptors can also be activated to provide analgesia (19). Our findings aligned with those of Unlugenc et al. (20), who investigated the impact of administering intravenous dexmedetomidine at a dose of 1 µg/kg 10 minutes prior to anesthesia induction. They discovered that this intervention significantly lowered the postoperative need for morphine. In a similar study, Sabry and Elmawy (21) compared dexmedetomidine and esmolol during cochlear implant surgery in children, finding that the time until the first request for analgesics was notably longer in the dexmedetomidine group. Moreover, Taghinia et al. (22) found that dexmedetomidine was effective in decreasing the need for postoperative analgesia. According to another study by Celebi et al., intravenous esmolol infusion decreased the need for analgesics during and after surgery, which in turn decreased VAS (23). Our findings align with those of Kol et al. (24), who investigated controlled hypotension using desflurane in conjunction with esmolol or dexmedetomidine during tympanoplasty in adults. They reported that the use of esmolol was linked to significantly shorter extubation and recovery times. However, it is noteworthy that Kol et al. also observed that recovery from anesthesia was considerably quicker in the esmolol group compared to the dexmedetomidine group (24).

### 5.1. Limitations

Our limitations include a small sample size, a single-center study with short-term assessment for immediate complications. Our recommendations include multi-center trials, including larger and more diverse patient populations, and extending follow-up periods to assess long-term outcomes.

### 5.2. Conclusions

Our research findings indicated that both dexmedetomidine and esmolol are safe and effective in enhancing surgical conditions and creating a dry surgical field, which improves visibility and shortens the procedure time during FESS. Dexmedetomidine contributed to a delay in the first request for analgesics and provided superior postoperative sedation, while esmolol was associated with quicker recovery.

## Data Availability

The dataset presented in the study is available on request from the corresponding author during submission or after publication. The data are not publicly available due to patient privacy

## Clinical Trial Registration Code:

NCT05703048 at the date of 01/10/2023.
